# A Retrospective Study of Epistaxis–ED in Bielsko-Biala, Poland

**DOI:** 10.3390/jcm14207314

**Published:** 2025-10-16

**Authors:** Maciej B. Hajduga, Katarzyna Kubalanca-Kwiecien, Wojciech Szczerbowski

**Affiliations:** 1 Department of ENT and Head & Neck Surgery, Provincial Hospital, Al. Armii Krajowej 101, 43-300 Bielsko-Biala, Poland; wszczerbowski@gmail.com; 2 Faculty of Health Sciences, University of Bielsko-Biala, Willowa 2, 43-309 Bielsko-Biala, Poland; 3 Saint Luke’s Hospital, Bystrzanska 94B, 43-309 Bielsko-Biala, Poland; kasiakubalanca@gmail.com

**Keywords:** epistaxis, season, age, emergency

## Abstract

**Objectives**: This research presents a retrospective analysis of cases of epistaxis in patients arriving at the Emergency Department (ED ENT) of the Provincial Hospital in Bielsko-Biala throughout 2015–2019. **Methods**: The analysis covered two periods: a general dataset (2015–2018) and a detailed clinical subset (2018–2019). Each case of a patient presenting with epistaxis, who was diagnosed with ICD-10 R04.0 Epistaxis upon admission to the ED, was analyzed in relation to the patient’s age and place of permanent residence. Moreover, the patients admitted from July 2018 to June 2019 were subjected to an in-depth analysis, which took into account their blood pressure, the use of anticoagulants or antiplatelet medication, upper respiratory tract infection (URTI) symptoms, deviated nasal septum, the type of dressing used, and potential trauma or nasal surgery. **Results**: A clear seasonality was observed when it comes to the number of patients reporting to the ED—there was a significant decrease in the number of patients with nasal bleeding during summer months. Patients aged over 60 years constituted the largest group. The vast majority of patients were diagnosed with a deviated nasal septum (ICD-10 J34.2). URTI symptoms were reported in a minority of patients (7%). Elevated blood pressure was more common among older patients. **Conclusions**: The risk of nose bleeding increases with age, hypertension, use of anticoagulant medication, and during winter months.

## 1. Introduction

Spontaneous nosebleeds and their appropriate treatment are one of the essential elements of emergency medicine. This condition occurs in all age groups but is most common among the elderly worldwide [1,2]. The symptom of bleeding is quite irritating alone, and, provided that it does not subside on its own, patients often seek medical help.

In order to implement effective methods of prophylaxis against epistaxis and reduce the number of patients requiring emergency care, factors that predispose to epistaxis must be analyzed [3,4]. It seems appropriate to analyze the cases in a given country, region or specific hospital [5].

Hypertension is widely believed to be one of the main factors contributing to epistaxis [6]. This condition may remain undiagnosed in some people [7]. For this reason, controlling and lowering blood pressure is one of the principal conditions for effective treatment [8]. Another important factor is the use of anticoagulants. It has been confirmed that especially older types of medications significantly increase the incidence of epistaxis [9]. Medications applied topically to the nasal cavities, such as steroids, may also be significant—albeit to a small degree [10].

The season, humidity, place of residence, geographical location and atmospheric factors are highly significant factors affecting the potential occurrence of epistaxis and possible arrival at the ED. A substantial number of papers have been devoted to this subject [11,12,13,14]. Most researchers describe winter as the season predisposing to epistaxis. This may be related to the fact that during winter, patients spend their time indoors more often, where central heating dries up the air. This hypothesis, however, is not confirmed by some researchers, who have claimed that in some regions no correlation exists between humidity and the incidence of epistaxis [15].

Therefore, it seems reasonable that cases of epistaxis should be analyzed in various geographical regions, countries, provinces or cities. In this way, it will be possible to learn and determine additional factors that may predispose patients to this ailment.

This study presents a retrospective analysis of epistaxis cases in patients reporting this ailment to the ED of the Provincial Hospital in Bielsko-Biala (Poland) from January 2015 to June 2019.

## 2. Materials and Methods

Bielsko-Biala is a county seat city located in the southern part of Poland. Its population is about 170,000 people. Bielsko-Biala County has about 160,000 inhabitants. The Provincial Hospital is the only medical center in the county maintaining a constant round-the-clock duty, including otorhinolaryngology. On average, the ED provides treatment for 45,000 patients annually, out of whom approximately 400 are diagnosed with epistaxis.

This study analyzed the cases of patients with nose bleeding who were initially diagnosed with ICD-10 R04.0 Epistaxis upon their admission to the ED in the triage area. No distinction was made as to whether the patients came to the ED on their own or were transported there from the place of residence by emergency services that had not been able to help the patient in their place.

Two periods were analyzed separately: (I) January 2015—December 2018—general demographics and seasonality, and (II) July 2018—June 2019—detailed clinical factors.

1758 patients treated from January 2015 to December 2018 were analyzed, and variables such as age and place of residence were analyzed.

Additionally, the number of patients in a given month was established.

The in-depth analysis was based on 314 cases of patients with epistaxis treated from July 2018 to June 2019. Apart from their age and place of residence, the following factors were also analyzed:

– Patient’s gender;
– Blood pressure above 150/90 mmHg;
– Whether the bleeding subsided on its own after the blood pressure dropped below 150/90 mmHg;
– Whether the patient took anticoagulants;
– Whether the patient had symptoms of URTI;
– Whether the patient had a deviated nasal septum;
– Whether it was the patient’s first or subsequent bleeding episode;
– Whether the bleeding was caused by an injury or whether the patient had recently had a nose/paranasal sinus surgery;
– Whether the epistaxis required an anterior or posterior nasal packing;
– Whether the treatment of epistaxis required hospitalization or surgery.

The term ‘deviated nasal septum’ refers to a clinically significant or obstructive deviation confirmed during ENT examination.

Hospitalized patients underwent endoscopic examination and, if required, local cauterization under general anesthesia. No sphenopalatine artery ligation was necessary.

### Statistical Analysis

Categorical variables were compared using the chi-square test. For trends across age groups and months, the chi-square test for trend was applied. Two-sided *p*-values < 0.05 were considered statistically significant. All analyses were performed using standard statistical software—Microsoft Excel 2021.

## 3. Results

Results are presented for two study periods: January 2015 to December 2018 and July 2018 to June 2019.

**Figure 1:**
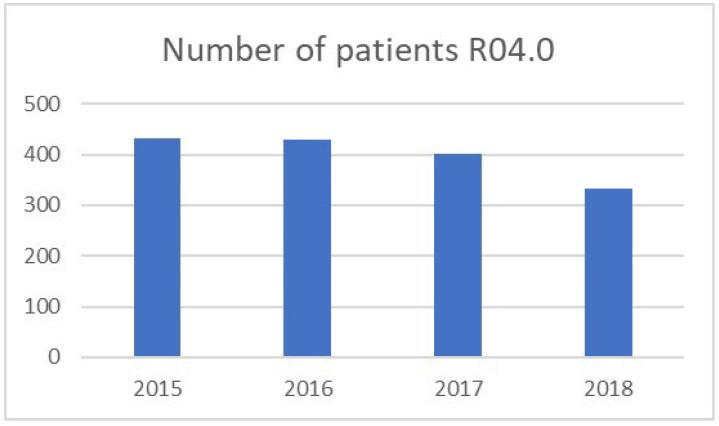
Number of patients with a diagnosis R04.0 Epistaxis.

Between January 2015 and December 2018, a total of 1758 patients arrived at the ED, for whom the main ICD-10 diagnosis in the triage area was R04.0 Epistaxis. From 2015 onwards, a downward trend could be observed in the number of patients in a given year (Figure 1).

**Figure 2:**
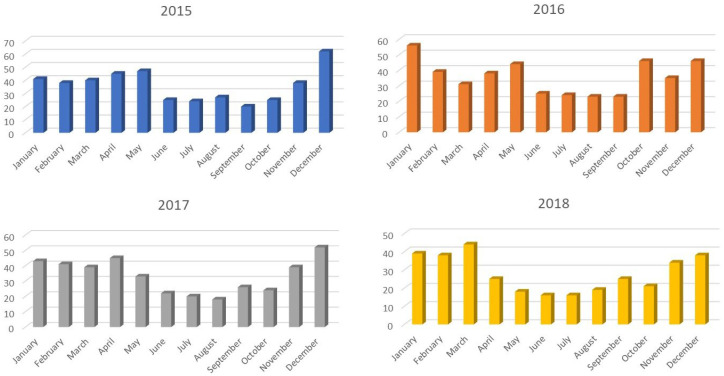
The number of patients in individual months throughout the years 2015 to 2018.

The “Seasonality” factor was also analyzed by counting the number of patients in a given month (within a given year) and comparing the obtained data. A significantly higher number of patients was observed from November to May. This tendency persisted every year (Figure 2).

**Figure 3:**
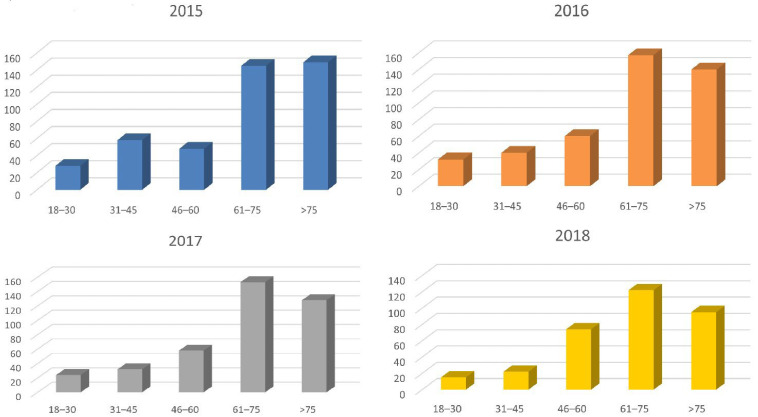
The number of patients in a particular age range during the years 2015 to 2018.

The age structure of patients was divided into age groups of 18–30, 31–45, 46–60, 61–75 and above 75 years old. Based on the number of patients in a particular age group, it was estimated that the risk of epistaxis increases significantly above the age of 60 (*p* = 0.008) (Figure 3). The tendency in the number of patients in the given age groups remained at a similar level each year.

**Figure 4:**
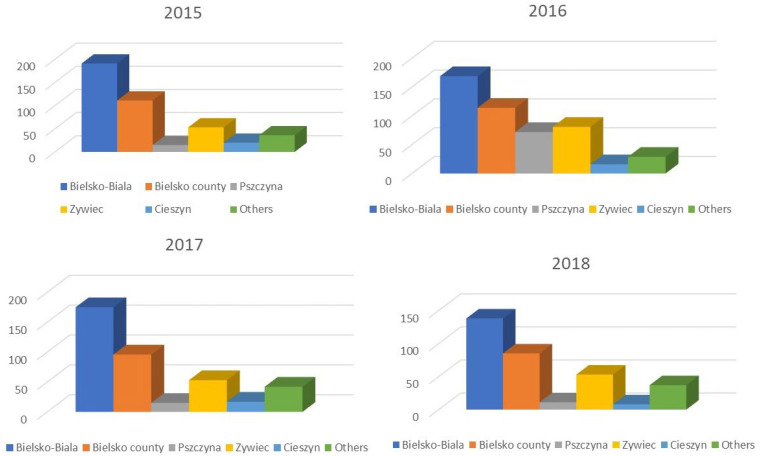
Number of patients from particular areas of residence during the years 2015 to 2018.

The overwhelming number of patients reporting to the ED came from the city of Bielsko-Biala. Despite a very similar population size of the city and county, significantly fewer patients came from the out-of-town area (*p* = 0.003). Additionally, a certain number of patients from neighboring cities and counties (Pszczyna, Zywiec, Cieszyn and others) were observed (Figure 4).

**Figure 5:**
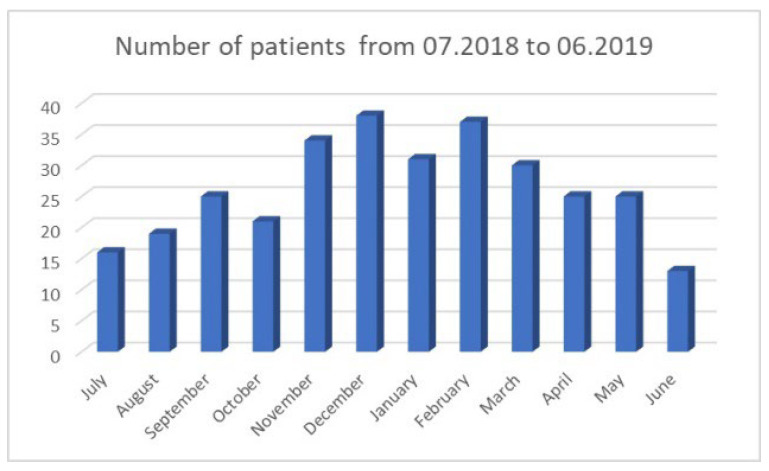
Number of patients with epistaxis in a given month.

**Figure 6:**
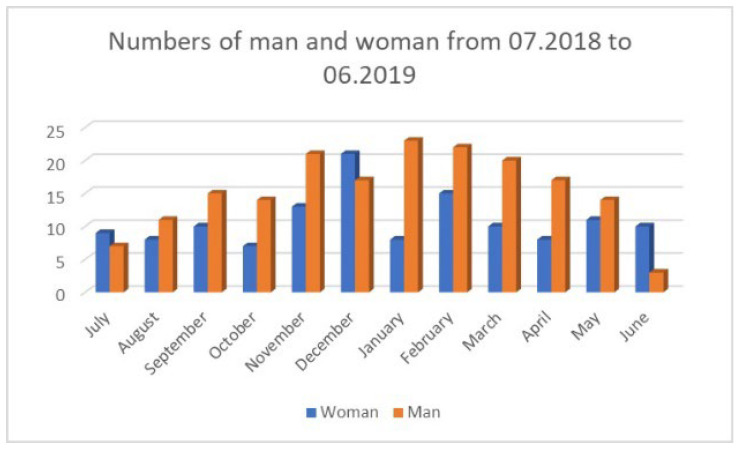
Number of patients with epistaxis by gender.

The cases of patients arriving at the ED because of epistaxis were analyzed in detail from July 2018 to June 2019 and a total of 314 patients were observed. The numbers of patients arriving each month are presented in Figure 5, with division by gender shown in Figure 6. There was a significant number of men attending (*p* = 0.0001)

Patients with a blood pressure of 150/90 mmHg and above constituted about 28% (*p* = 0.0004) of the total number (88 of 314) of patients.

In patients with epistaxis and elevated blood pressure, one of the first rescue actions is to lower this value. Only 8% of patients experienced idiopathic resolution of epistaxis after BP reduction below 150/90 mmHg and no further local nasal cavity dressing was needed.

It was also observed that 32% of patients from the group undergoing detailed analysis suffered from recurrent epistaxis, while the remaining part had reported to the ED immediately after the first onset of this symptom.

Patients using anticoagulants and antiplatelet agents accounted for approximately 33% of those attending the ED with epistaxis. During the interview, patients often did not remember exactly what kind of drugs they were using. Therefore, these types of drugs are generally referred to as “reducing blood clotting”.

During otolaryngologic examination, up to 84% of patients had nasal septum deviation. Patients with features of upper respiratory tract infection constituted only about 7% of the total number of patients.

Patients after nasal injury, which led to epistaxis requiring dressing, constituted 3%, while the patients who had undergone nasal and sinus surgery within 2 weeks before attending the ED constituted 2%.

In about 88% of patients, classical frontal nasal packing was an effective dressing. Among the remaining patients, 11% did not need a nasal dressing because bleeding stopped idiopathically after controlling the cause of direct bleeding, such as lowering high blood pressure. Only the nasal cavities were cleaned of blood clots.

Only in the case of about 2% of patients was hospitalization at the ENT ward required because bleeding could not be suppressed in ED conditions. Despite the posterior nasal packing, it was necessary to apply hospital treatment and to observe the patient or conduct a surgical procedure that contained and dressed the bleeding. Significantly more patients with epistaxis were received between November and May. Elevated blood pressure was significantly more frequent among patients aged ≥ 60 years (*p* < 0.05).

## 4. Discussion

A decreasing trend in the number of patients with epistaxis was observed over 4 years, where slightly over 400 patients were received in 2015 and around 300 in 2018. Out of all patients reporting to the ED, only about 0.89% were patients with epistaxis. An average of 1.2 patients per day. A lower index, 0.46% in the United States, is described by Seidel D. U. et al. [16]. Bray D. et al. indicate six patients daily with epistaxis [17] in the analysis of admissions to Scottish hospitals in 1994–2005. They also describe the correlation between the number of patients with epistaxis and the moon phase. A similar phenomenon is probably observed at the ED of the Provincial Hospital in Bielsko-Biala, where, subjectively, patients with epistaxis are received more often, during, e.g., a period of significant decrease in atmospheric pressure, than on other days. However, this correlation is not described in the test results because it has not been analyzed. Seidel D. U. et al. [16] obtained similar results regarding the age of patients and the season of the year. There was a significant number of patients with epistaxis who were over 70 years old, and most cases of epistaxis occurred in the winter months, while the number decreased during the summer. In our work, we observed a significant increase in the number of patients with epistaxis who were already over 60 years old.

From November to May, there was a significant increase in the number of patients, with a decrease during the summer months. Many researchers observe a very similar tendency in other countries and geographical regions [18,19]. However, some of them do not confirm this dependence [20]. It can be assumed that in areas where the average winter temperature is much lower than in the summer months, there is a need for heating indoor premises, which in turn causes a decrease in the humidity of the inhaled air, drying the nasal mucosa and, as a consequence, leading to epistaxis. A significant difference in the temperature indoors and outdoors might be an additional factor.

Each year, a difference was observed in the number of patients from the city compared to the county, despite a similar population size in the city of Bielsko-Biala and Bielsko-Biala County. Significantly more patients living in the city were received. This may be caused by the shorter distance from the hospital to be covered by patients from the city who, on account of ease and duration of the journey, arrived more often, while patients from the county (out-of-town) area probably tried to cope with epistaxis alone, and it was effective.

Epistaxis affected men significantly more often than women (Figure 6). This is a tendency described also by other researchers [21].

In about 28% of patients with epistaxis, a blood pressure of 150/90 mmHg and above was observed. This correlation is confirmed by other researchers, although it ranges between about 20% and 50%. Reis L. R. et al. indicate in their studies that patients with epistaxis are more likely to have increased blood pressure values compared to other ED patients. In this regard, research is conducted in two directions. The incidence of epistaxis in patients with arterial hypertension is analyzed [18], and, similarly to our work, the number of patients with arterial hypertension can be analyzed among patients with epistaxis [19]. Importantly, approximately 8% of patients with increased blood pressure and epistaxis did not require surgical dressing. The bleeding stopped idiopathically after blood pressure normalized. The most commonly administered and effective drug in the ED is captopril (angiotensin-converting-enzyme inhibitor) at a dose of 25 mg, received sublingually. Elevated BP values recorded at admission may reflect stress-related transient hypertension rather than a chronic condition.

It was observed that about 32% of patients attending the ED suffered from recurrent bleeding, which they could not cope with at home, while the remaining group (about 68%) reported due to the first epistaxis incident.

About 33% of patients used anticoagulants. The convergence of their use with epistaxis is confirmed by other researchers. P. Gomez et al. describe the relationship between the use of anticoagulants and the increased risk of epistaxis [20]. Other researchers confirm this relationship [22]; however, when using modern anticoagulants, the risk of bleeding decreases [23].

Approximately 84% of patients had nasal septum deviation during ENT examination. However, it is difficult to find a significant correlation between deviated nasal septum (DSN) and epistaxis, as no control group without epistaxis has been studied. It is important, however, that the patient with DSN is more difficult to dress, especially when bleeding from the posterior part of the nose. The study did not include a control group; therefore, the association between DSN and epistaxis cannot be considered causal.

Patients with symptoms of URTI constituted about 7%. However, URTIs cannot be correlated in any way with a higher risk of epistaxis, as patients often use nasal decongestant drugs such as xylometazoline hydrochloride or cause mechanical damage to the mucous membrane by clearing the nasal cavities.

Patients with nasal injuries (3%) and patients after nasal and sinus surgery (2%) accounted for a relatively small percentage.

At the ED of the Provincial Hospital in Bielsko-Biala, the basic method of dressing is the classic frontal nasal packing. Among the analyzed patients, 88% of the cases were effective. Although many researchers indicate modern solutions as equally effective [24], classic nasal packing is economically justified and is often a very good solution for patients with massive nasal septum deviation. Balloon or sponge devices commonly used in the U.S. were not employed due to cost considerations and because ENT specialists manage all epistaxis cases directly. Classical anterior packing remains our standard and effective approach.

About 11% of patients did not require mechanical treatment. In most of these cases, normalizing blood pressure was enough.

2% of patients with epistaxis also required hospitalization at the ENT department, apart from the possible dressing with a posterior nasal packing. This was most often caused by the necessity of a blood transfusion, intensive treatment of arterial hypertension or intravenous drug administration.

Information on systemic comorbidities such as diabetes or liver disease was not consistently available in the retrospective dataset, which we acknowledge as a study limitation.

## 5. Conclusions

Patients with epistaxis represent a small percentage of patients received by the ED. However, they require specialized dressing. The ED of the Provincial Hospital in Bielsko-Biala is highly effective at attending to such cases. A significant relationship between the season of the year and the number of patients with epistaxis was confirmed. The ED is visited by a relatively higher number of patients who live closer to the hospital compared to patients from the out-of-town county area. Through the implementation of preventative education, blood pressure measurements or controlling the administration of anticoagulants by general practitioners, it is possible to reduce the number of patients with epistaxis at the ED, thus obtaining significant savings.

## Data Availability

The data are available on request from the corresponding author. The data are not publicly available due to patient privacy restrictions.

## Abbreviations

The following abbreviations are used in this manuscript:

ED	Emergency Department
ICD-10	International Classification of Diseases, 10th Revision
URTI	Upper Respiratory Tract Infection
DSN	Deviated Nasal Septum
BP	Blood Pressure
ENT	Ear, Nose and Throat (Otolaryngology)
ACE inhibitor	Angiotensin-Converting Enzyme Inhibitor
